# Probe-free label system for rapid detection of *Cronobacter* genus in powdered infant formula

**DOI:** 10.1186/s13568-018-0689-x

**Published:** 2018-09-29

**Authors:** Shiqian Fu, Yujun Jiang, Xia Jiang, Yueming Zhao, Sihan Chen, Xinyan Yang, Chaoxin Man

**Affiliations:** Key Laboratory of Dairy Science, Ministry of Education, College of Food Science, Northeast Agricultural University, Harbin, 150030 China

**Keywords:** Loop-mediated isothermal amplification, Probe-free label based lateral flow assay, *Cronobacter* species, Powdered infant formula

## Abstract

*Cronobacter* species previously known as *Enterobacter sakazakii* poses high risks to neonates and infants. In this work a rapid detection method was developed which combined loop-mediated isothermal amplification with lateral flow assay for detection of *Cronobacter* species in powdered infant formula. The fast amplification reaction without betaine was established and capable of performing DNA replication within 25 min. Based on the novel probe-free labeling methods, we established a lateral flow assay to capture the specific loop-mediated isothermal amplification amplicons which were labeled with fluorescein isothiocyanate and biotin. And the final detection time of this system was within 40 min. The false positive results of the lateral flow assay induced by primer dimer tagged with fluorescein isothiocyanate and biotin were eliminated by Taq single strand DNA binding protein (4 ng/μL). Simultaneously, the efficiency of the fast loop-mediated isothermal amplification assay was achieved. By injection of Taq SSB into the amplification assay as a replacement for betaine, the novel probe-free method could detect *Cronobacter* species with high specificity and sensitivity at the detection limit in PIF of 10^1^ cfu/g. Our overall strategy has excellent potential in the rapid diagnosis of *Cronobacter* species label-free by integrating loop-mediated isothermal amplification and lateral flow assay.

## Introduction

*Cronobacter* species (*Cronobacter* spp.) isolated from plant-based food products, previously known as *Enterobacter sakazakii*, are foodborne pathogens that pose a high risk of infection to neonates as well as immuno-compromised individuals causing meningitis, necrotizing enterocolitis, and bacteremia (Susan and Forsythe 2011), and the fatality rate is about 40–80% (Yan and Fanning 2015). Meanwhile, all *Cronobacter* spp., except *C. condimenti*, have been associated with human infections (Cruz-Córdova et al. 2012). It has been reported that the powdered infant formula (PIF) is the main source of their infection (Song et al. 2016). And recent research based on international microbiological standards suggested that all species of *Cronobacter* must be absent in 10 grams of PIF (Odeyemi and Sani 2016). In addition, WHO (2007) has classified *Cronobacter* together with *Salmonella* as group A pathogens associated with PIF with clear evidence of illness in infants. Therefore, appropriate technologies to monitor *Cronobacter* spp. contaminated raw materials and products, especially in PIF is of great significance (Gautam et al. 2015).

The traditional cultural and biochemical-based methods are tedious and require skilled personnel and time (Blažková et al. 2009). Thus rapid methods are viewed as alternative means to detect and monitor *Cronobacter* spp. Nucleic acid amplification methods including PCR (Moraes and Maruniak 1997; Chen et al. 2015), multiplex PCR (Gordon et al. 2015; Zhang et al. 2015), real-time PCR (Cecilia et al. 2015; Mai et al. 2015) have attracted the attention of a plethora of engineers and scientists due to their relatively specific and accurate characteristics (Tafelski et al. 2015), but the limitations of their thermal cycling, complicacy and low level sensitivity impedes its applicability in rapid and routine monitoring of pathogens (Cornelissen et al. 2016). An easy method to operate loop-mediated isothermal amplification (LAMP) developed by Notomi in 2000 showed attractive potential and sufffered the problems common in complicated reactions with the inhibitory components extracted from the crude samples (Notomi et al. 2000). Recent years, LAMP has been successfully applied to detection for pathogens, and a simple heating device has been employed at a fixed temperature with an typical amplification time of 40–60 min to produce large amounts of DNA to obtain a detection limit of 10^1^–10^2^ cfu/g by agarose gel electrophoresis (AGE) analysis (Hu et al. 2009; Fan et al. 2012).

To detect the LAMP products, lateral flow assay (LFA) have been well used as a replacement for carcinogenic ethidium bromide (EB) and inaccurate dyes due to its rapid, convenient, visual and accurate features (Wang et al. 2013; Chen et al. 2016). Previously, the LAMP amplicons were obtained at a stable temperature, hybridized with the fluorescein isothiocyanate (FITC)-labeled probe based on the base-pairing rule and developed the results on the LFA (Ding et al. 2010; Wang et al. 2013). Then amplicons double labeled with FITC/biotin were captured by streptavidin (SA)-tagged gold nanoparticles (AuNPs) which appeared visible band in the T line of the strip. Thus, the LAMP-LFA method based on probe-hybridization was successfully applied in bio-diagnostics area. Simultaneously, the complicated instruments were not needed and the detection results could be observed in a short time.

Nevertheless, the utilization of FITC probe induces nonspecific amplification and generates false positive results. Therefore, it is necessary for a label-based system to eliminate probe-hybridization. Recently, a novel probe-free label system has been reported in which the double labeled FITC/biotin LAMP amplicons captured by AuNP-based assay are visible in the LFA (Najian et al. 2016; Zhang et al. 2015). However, the 4–6 sets of LAMP primers increase the chances of obtaining spurious amplification products primarily because of the formation of the primer dimers that consumed the reaction components. The pioneer work by Brownie et al. first employed a sequence of additional nucleotides (a Tail) at the 5′ ends of amplimers to eliminate the primer dimers in PCR (Brownie et al. 1997), in which the work they did complicated the simplicity of the detection system and increased the risk of false results due to improper design. While some investigations have been done by using the single strand DNA binding (SSB)-like protein to resolve the problem in PCR assay (Nimitphak et al. 2008; Tian et al. 2014). Furthermore, the thermally stable Taq SSB protein enables to selectively combine the protein with primers and reduce or eliminate the dimer formation, thus it can be applied in the LAMP assay to obtain the specific products and enhance the efficiency of amplification. Taken together, we considered to establish a novel label based LAMP-LFA, expecting the Taq SSB protein can be efficiently used for the *Cronobacter* spp. detection in PIF.

Given the importance of the safety of PIF, a novel label based LAMP-LFA system was developed which was rapid and efficient to detect the target DNA of *Cronobacter* spp. from contaminated PIF within 40 min. In this investigation, the specific primers designed based on the *ITS* gene were used to perform the LAMP reaction. Meanwhile, the specific “Fast LAMP” amplicons were achieved by the addition of Taq SSB protein to enhance the amplification efficiency and provide a novel method for rapid detection of *Cronobacter* spp. in the protein-rich PIF.

## Materials and methods

### Bacterial strains, DNA extraction

19 *Cronobacter* strains were utilized for specificity assays which containing 13 *C. sakazakii* strains (including ATCC 29544, BAA-894, NCTC 8155, SP 291, CE 13, CE 15, CE 16, CE 28, CE 29, CE 32, CE 52, CE 55 and CE 56) and 6 non-*C. sakazakii* strains. Simultaneously, 29 non-*Cronobacter* strains were used in this study (Table 1). *C. sakazakii* ATCC 29544 was used as sample to test the sensitivity in pure culture. A single clone of *C. sakazakii* ATCC 29544 picked from a TSA plate was incubated at 37 °C for 12 h in TSB (TSB, TSA without agar). After incubation, the DNA boiling extraction condition was determined. 1 mL of the *C. sakazakii* culture was centrifuged at 12,000 rpm for 1 min (*C. sakazakii* collection), then the pellet was resuspended in TE_1_ buffer (10 mM Tris, 1 mM EDTA, pH = 8.0) and boiled at 95 °C for 6 min. Subsequently, the pellet was placed on ice for 7 min and then centrifuged at 4000 rpm for 3 min. The supernatant was removed to a clean tube and stored at − 20 °C for further use.

**Table 1:** Bacterial strains used in this investigation and the detection results of the specificity

No.	Bacterial species	Strain	AGE	LFA
	*Cronobacter* strains			
1	*C. sakazakii*	ATCC 29544	+	+
2	12 *C. sakazakii*	Laboratory	+	+
3	*C. malonaticus*	DSM 18702	+	+
4	*C. turicensis*	DSM 18703	+	+
5	*C. muytjensii*	ATCC 51329	+	+
6	*C. universalis*	NCTC 9529	+	+
7	*C. dublinensis*	DSM 18705	+	+
8	*C. condimenti*	LMG 26250	+	+
	Non-*Cronobacter* strains			
9	*Enterobacter cloacae*	ATCC 3503	−	−
10	*Enterobacter aerogenes*	ATCC 13048	−	−
11	*Staphylococcus aureus*	CMCC 26075	−	−
12	*Staphylococcus aureus*	ATCC 13565	−	−
13	*Staphylococcus epidermidis*	ATCC 26069	−	−
14	*Staphylococcus xylosus*	ATCC 29971	−	−
15	*Listeria monocytogenes*	CMCC 54004	−	−
16	*Listeria monocytogenes*	CMCC 54006	−	−
17	*Listeria monocytogenes*	CMCC 54002	−	−
18	*Listeria innocua*	ATCC 33090	−	−
19	*Listeria welshimeri*	ATCC 43548	−	−
20	*Listeria grayi*	CICC 21670	−	−
21	*Escherichia coli O157:H7*	ATCC 25922	−	−
22	*Salmonella typhimurium*	ATCC 14028	−	−
23	*Salmonella enteritidis*	ATCC 13076	−	−
24	*Salmonella choleraesuis*	CMCC 50018	−	−
25	*Salmonella* *dublin*	CMCC 50092	−	−
26	*Salmonella paratyphi A*	CMCC 50093	−	−
27	*Salmonella enterica paratyphi B*	CMCC 50094	−	−
28	*Salmonella* *arizonae*	CMCC 47020	−	−
29	*Salmonella* *thompson*	CMCC 50120	−	−
30	*Salmonella* *pullorum*	ATCC 9120	−	−
31	*Pseudomonas putida*	CGMCC 1.1819	−	−
32	*Pseudomonas aeruginosa*	ATCC 27853	−	−
33	*Pseudomonas* *fluorescens*	CGMCC 1.1802	−	−
34	*Bacillus* *cereus*	CMCC 63303	−	−
35	*Shigella* *flexneri*	CMCC 51572	−	−
36	*Vibrio parahaemolyticus*	ATCC 17802	−	−
37	*Vibrio parahaemolyticus*	ATCC 33847	−	−

ATCC, American Type Culture Collection; DSM, Deutsche Sammlung von Mikroorganismen und Zellkulturen; NCTC, National Collection of Type Cultures; LMG, Belgian Coordinated Collections of Microorganisms; CMCC, National Center for Medical Culture Collections; CICC, China Center of Industrial Culture Collection; CGMCC, China General Microbiological Culture Collection; AGE, agarose gel electrophoresis; LFA, lateral flow assay; +, positive result; −, negative result

### Fast LAMP assay

#### Target gene and primers

LAMP primers shown in Table 2 were designed based on the conserved sequence of the *Cronobacter* spp. *ITS* gene (GenBank: AY702093) which described previously (Hu et al. 2009). A set of 6 primers including 2 outer primers (F3 and B3), 2 inner primers (FIP and BIP) and 2 loop primers (LF and LB) which can accelerate the LAMP reaction. All the primers were synthesized commercially by Invitrogen (Shanghai, China).

**Table 2:** The sequence of LAMP primers designed based on the *ITS* gene of *Cronobacter* spp.

Primer	Sequence (5′–3′)
ITS-F3	AAATGCGCGGTGTGTCAG
ITS-B3	GGTTTCCCCATTCGGACAT
ITS-FIP	ACCGTGTACGCTTGTTCGCTTTCTCTCAAACTCGCAGCAC
ITS-BIP	GGCAGTCAGAGGCGATGCGCCGGTTATAACGGTTCA
ITS-LF	AACCTCACAACCCGAAGA
ITS-LB	AGCGCCGGTAAGGTGATA

#### Optimization of LAMP reaction time

The typical LAMP reaction assay was performed in 8 durations (0, 5, 10, 15, 20, 25, 30, 35, 40 min) to generate amplicons to determine the minimum detection time targeted at different concentrations of *Cronobacter* spp. bacteria. The amplified products were analyzed by AGE. The shortest amplification time was used to optimize the LAMP amplification assay including the concentration of dNTP, the ratio of the primers and the amplification temperature using Bst 2.0 DNA polymerase. DNA template was replaced with nuclease-free water in the negative control reaction. 2% AGE stained with EB and the LFA were used to analyze the LAMP products, respectively.

### Lateral flow assay

#### Preparation of AuNPs-antibody complex

AuNPs (mean diameter, 20 nm) were prepared according to the previous method (Rivas et al. 2015). The pH of the AuNPs suspension was adjusted to 8.3 with 0.2 M K_2_CO_3_ (2 μL). 3 μL of the anti-FITC antibody (100 μg/mL) obtained from Santa Cruz Biotechnology was added into the 1 mL of the adjusted colloidal gold solution with gentle stirring in dark for 15 min. Then 10% BSA was added to the solution in dark for another 15 min to ensure that the colloidal gold nanoparticles were combined with the antibody and the free of gold nanoparticles were sealed. Subsequently, the mixtures were centrifuged at 10,000 rpm for 15 min. After centrifugation, the pellet was suspended in 100 μL blocking buffer (10 mM Tris, 1% BSA, 2% trehalose, pH = 8.0) and stored in a dark bottle at 4 °C until use. The AuNP-antibody complex was used in the LFA.

#### Preparation of LFA

**Fig. 1:**
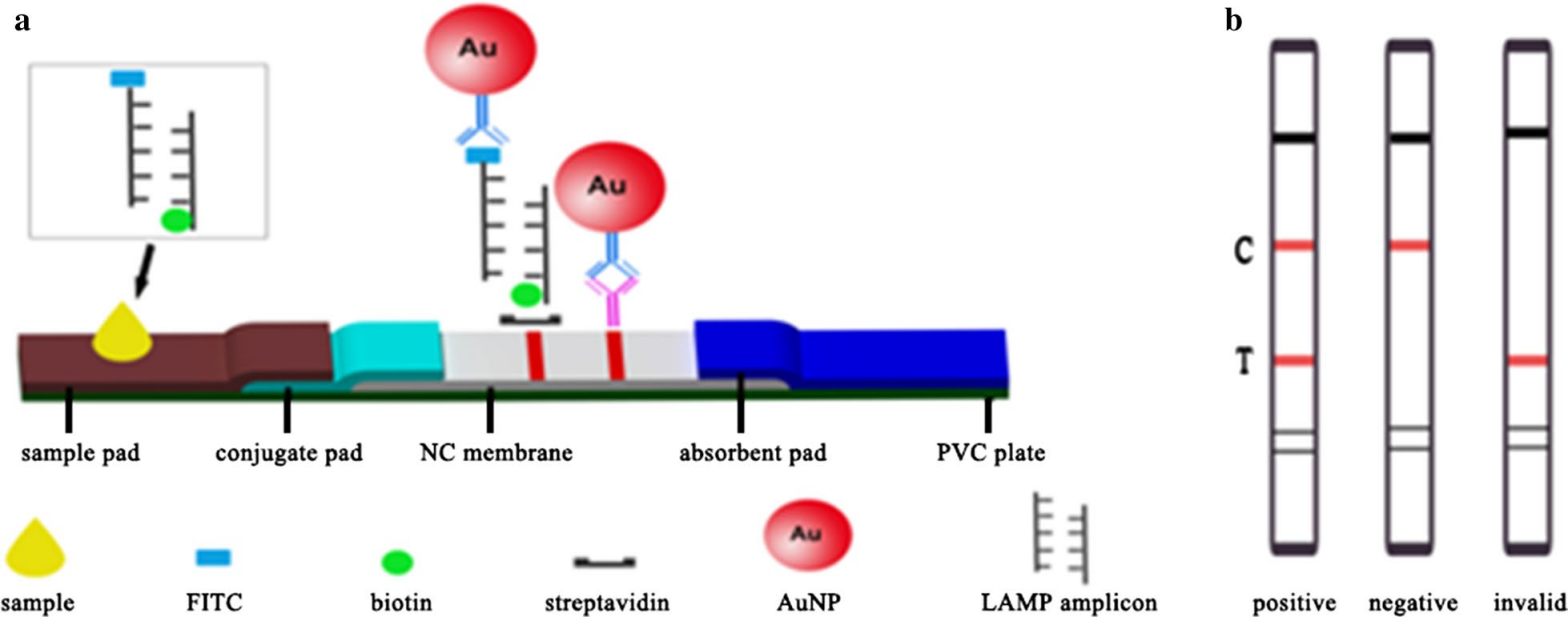
Schematic diagram of the lateral flow assay. **a** Schematic view of lateral flow assay and the detection principle; **b** illustration of LFA test results

The composition of LFA which contained five parts (including PVC plate, sample pad, conjugate pad, absorbent pad, nitrocellulose (NC) membrane) obtained from JieYi Biotechnology was demonstrated in Fig. 1a. The sample pad was saturated with running buffer (100 mM Tris, 0.2% BSA, 0.05% casein, 4% trehalose, 0.5% Tween 20) for 10 min, then dried at 42 °C for 1 h and stored in the refrigerator with desiccants at 4 °C for future use. The SA obtained from Sigma-Aldrich diluted by PB (0.01 M, pH = 7.4) to 2.5 mg/mL and goat anti-mouse IgG (1 mg/mL) purchased from Bioss (Beijing, Chian) were immobilized onto the NC membrane with a volume of 1 μL/cm to form the T and C lines, respectively. Pure cellulose fiber was used as the absorbent pad. The strips were cut into 4 mm width and stored in a desiccator at 4 °C for the detection of *Cronobacter* spp. ZX1000 Dot Dispensing Plat form XYZ3050 (BioDot, USA) and Guillotine Cutter HGS201 (Autokun, China) were used to dispense reagents and cut the strips, respectively.

#### Colorimetric detection of the LAMP amplicons with LFA

The LAMP amplicons labeled with FITC (incorporated with the FIP primers) and biotin moieties (incorporated with the BIP primers) interact with the AuNP–antibody and SA, respectively (Fig. 1a). Then the LFA was inserted into the samples which contained 5 μL of the obtained “Fast LAMP” amplicons and 100 μL of running buffer. The samples were absorbed by the sample pads capillary, then passed through the NC membrane, finally they were captured and realized visualization. After 10 min later, the results were shown in the strip, in which the test line was visible due to the binding of SA to biotin in the presence of the LAMP products and the control line become visible when the goat anti-mouse IgG antibodies recognized the excess antibodies binding with the AuNPs. The evaluation of results was provided in Fig. 1b.

### Primer dimer caused by probe-free label system

The LAMP primers [C(6, 2) = 15 combination modes] directly double labeled with FITC and biotin were used to test the non-specific amplification results in LFA. Briefly, all of the 15 combination modes used as negative controls without the DNA template were tested to assess self-dimerization and hetero-dimer formation. The FITC probe which conducted hybridization assay was used as the positive control with DNA template and the sequence was listed in Table 3. All of the 15 combination modes were analyzed by LFA and AGE to determine and check the self-dimerization and hetero-dimer formation.

**Table 3:** The sequence of FITC probe designed for labeling LAMP primer

Primer name	Sequence (5′–3′)
FITC probe	CTCAAACTCGCAGCACGAAGACT

### Effect of Taq SSB protein in LAMP assay

For eliminating the false-positive results due to non-specific amplification of primers, the Taq SSB protein obtained from Novoprotein (Shanghai, China) was introduced to the LAMP assay. The Taq SSB protein(0, 2, 4, 6, 8, 10 ng/μL)was infiltrated into the 25 μL amplification assay to test the effect for LAMP system and the analysis of results was performed by AGE. Besides, the novel label-based LAMP system was carried out in the absence and presence of Taq SSB protein to verify the effect of omitting the false-positive results using the double FITC and biotin directly labeled to the mispairing primers. Tests were performed in triplicates.

### PCR assay

The outer primers obtained from the LAMP primers F3 and B3 were used to perform PCR reaction and then tested for its specificity and sensitivity. The PCR reaction was conducted in a 25 μL reaction mixture containing: 2× PCR Master Mix (12.5 μL), 10 μM primer F3 and B3, DNA template (2 μL) and nuclease-free water up to 25 μL. The cycling profile was: initial denaturation at 94 °C for 3 min, 30 cycles of denaturation at 94 °C for 30 s, annealing at 61 °C for 45 s, and extension at 72 °C for 45 s, and a final extension at 72 °C for 5 min. Finally, the amplified 189 bp-PCR products were observed by 1% AGE stained with EB.

### Specificity test of LAMP-LFD

A total of 19 *Cronobacter* strains containing 13 *C. sakazakii* strains, 6 non-*C. sakazakii* strains and 29 non-*Cronobacter* strains were used to evaluate the specificity of the LAMP assay. DNA templates (2 μL) obtained from the fresh overnight cultures were subjected to LAMP-AGE and LAMP-LFA to test the specificity. Tests were repeated in triplicates.

### Sensitivity test in pure culture and in PIF

The sensitivity in pure culture of LAMP assay was determined by using tenfold serial dilutions of the *C. sakazakii* strains. Aliquot of the 2 μL extracted DNA of tenfold serial dilutions (from 4.8 × 10^6^ cfu/mL to 4.8 × 10^−1^ cfu/mL) of the *C. sakazakii* ATCC 29544 were used as the template to perform the LAMP and PCR assay, respectively. And 2 μL of nuclease-free water was used as a negative control in place of DNA. Then the PCR and LAMP products were subjected to AGE and LFA for analysis. Sensitivity tests were repeated in triplicates.

To determine the detection limit of LAMP-LFA in PIF without enrichment, 25 g of PIF without *C. sakazakii* were homogenized in 225 mL PBS, and then 9 mL of the PIF culture was inoculated with 1 mL of tenfold serial dilutions of *C. sakazakii* (ATCC 29544) culture to obtain a level of contamination from 5.6 × 10^5^ to 5.6 × 10^0^ cfu/g. Subsequently, aliquot of 2 μL DNA extracted from *C. sakazakii* ATCC 29544 in PIF were subjected to PCR and LAMP assay, respectively. And 2 μL of nuclease-free water was used as a negative control in place of DNA. The sensitivity of the rest six *Cronobacter* species contaminated in PIF was tested according to the methods listed above. Then the PCR and LAMP products were subjected to LFA for analysis. Sensitivity tests were repeated three times.

## Results

### Establishment of “Fast LAMP” assay

As shown in Table 4, the amplicons obtained from the 25 min amplification reaction were visibly detectable for *C. sakazakii* at 10^0^ cfu/mL. Ultimately, the optimized “Fast LAMP” reaction mixture (25 μL) without betaine contained 1.4 μM each of primers FIP and BIP, 0.2 μM each of primers F3 and B3, 0.8 μM each of primers LF and LB, 8 U of Bst 2.0 WarmStart DNA polymerase, 2.5 μL of 10× ThermoPol Buffer [20 mM Tris–HCl, 10 mM KCl, 8 mM MgSO_4_, 10 mM (NH_4_)_2_SO_4_ and 0.1% Triton X-100], 2 mM dNTPs, 4 mM MgSO_4_ and 2 μL of template DNA. The optimized reaction conditions were performed in a water bath at 65 °C with the minimum of 25 min and terminated at 80 °C for 2 min.

**Table 4:** The sensitivity study of the LAMP assay to detect *Cronobacter* spp. at different time periods

Amplification time (min)	LAMP results							
	Colony forming unit of *Cronobacter* spp. (cfu/mL)							
	10^−1^	10^0^	10^1^	10^2^	10^3^	10^4^	10^5^	10^6^
0	−	−	−	−	−	−	−	−
5	−	−	−	−	−	−	−	−
10	−	−	−	−	−	+	+	+
15	−	−	−	−	−	+	+	+
20	−	−	−	+	+	+	+	+
25	−	+	+	+	+	+	+	+
30	−	+	+	+	+	+	+	+
40	−	+	+	+	+	+	+	+

+, positive result; −, negative result

### Primer dimer caused by probe-free label system

**Fig. 2:**
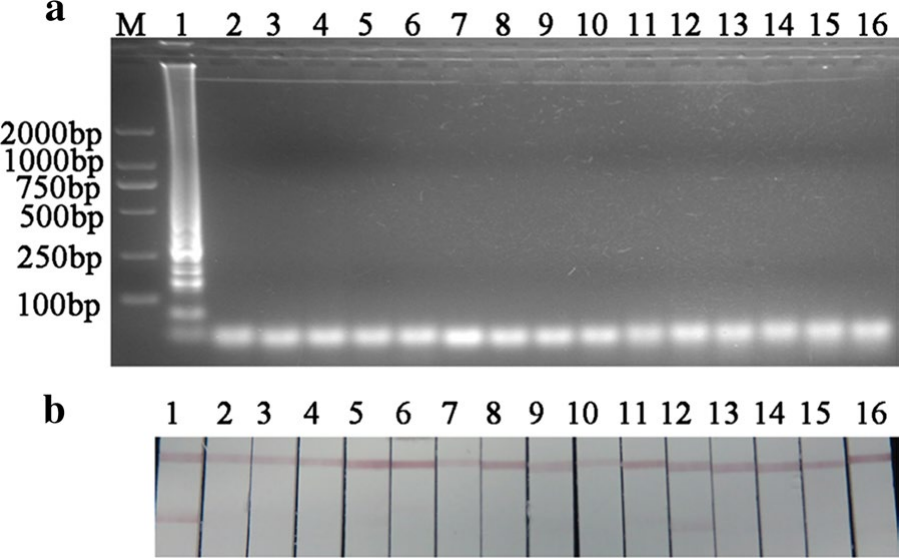
The analysis of the primer dimers’ location in loop-mediated isothermal amplification assay. **a** Agarose gel electrophoresis analysis; **b** lateral flow assay analysis

Using gel analysis the primers produced a stable and obvious incorrect < 50-bp primer dimer. Further we found that by AGE analysis all the 15 combination modes as the negative control without DNA templates gave the negative results and the probe-hybridization assay as the positive control with DNA template provided the positive result. Compared with AGE analysis, one combination mode of F3 and LF in LAMP assay as the negative control gave an unexpected positive result by LFA analysis (Fig. 2).

### Effect of Taq SSB protein in LAMP assay

**Fig. 3:**
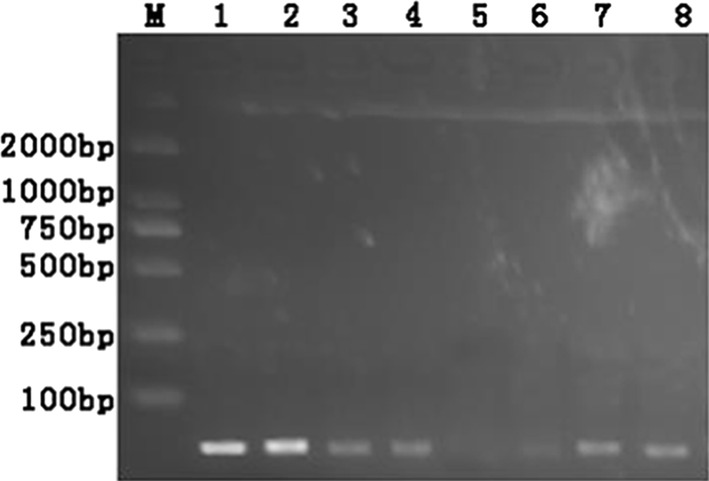
The optimization of Taq SSB protein. *M* marker. Lanes 1–8: the concentrations of Taq SSB protein ranging from 0 to 7 ng/μL. *SSB* single strand DNA binding

**Fig. 4:**
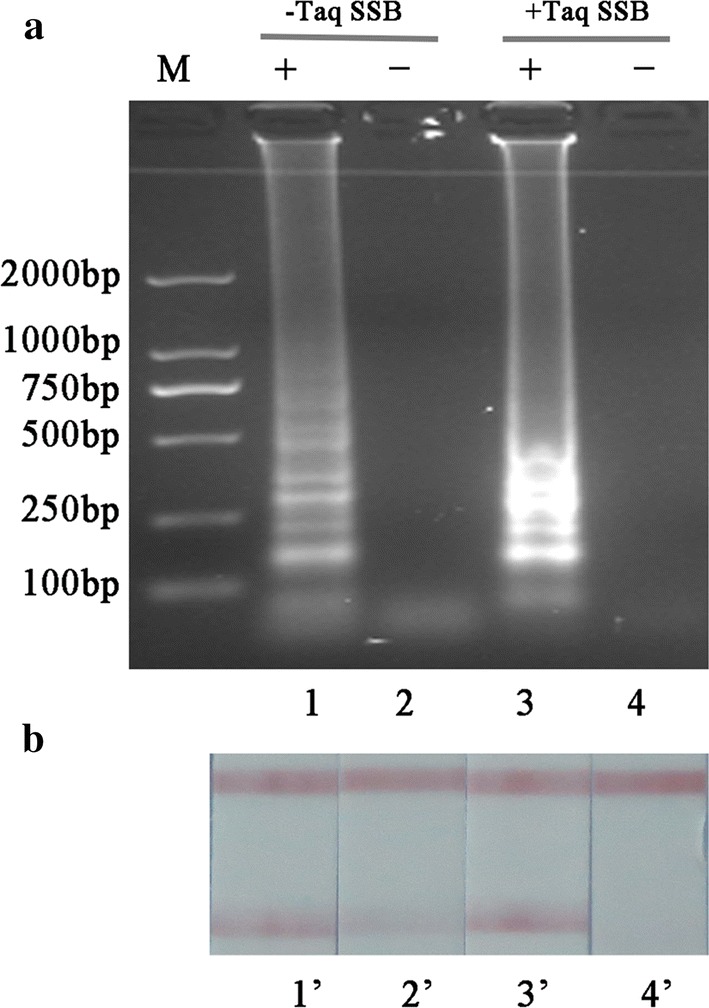
The analysis of the influence of Taq SSB protein in the LAMP reaction. **a** Agarose gel electrophoresis analysis; **b** lateral flow assay analysis. SSB = Single strand DNA binding

As shown in Fig. 3, it was found that the most suitable concentration of Taq SSB protein introduced into the LAMP assay was 4 ng/μL. Then the amount of LAMP primer dimer was evaluated by AGE analysis in the presence (4 ng/μL) or absence of Taq SSB protein. As shown in Fig. 4a (lane 1 and lane 2), a large concentration of dimer products was formed in the absence of Taq SSB protein. A different situation was observed in the presence of Taq SSB protein shown in lane 3 and lane 4 with a decrease in dimer products. In addition, by LFA tests the spurious results were ruled out in the presence of Taq SSB protein compared to results obtained in the absence of Taq SSB protein using the mispairing primers F3 and LF (lanes 3′ and 4′). The false positive results induced by primer dimer were efficiently eliminated.

### Specificity of LAMP-LFD assay

19 *Cronobacter* spp. strains and 29 non-*Cronobacter* spp. strains were used to test the specificity of LAMP by the double AGE and LFA methods. Results indicated that 19 *Cronobacter* spp. strains gave positive results and 29 non-*Cronobacter* spp. strains produced negative results by AGE and LFA analysis (Table 1). Simultaneously, the structures of the positive amplified products were confirmed by DNA sequencing and the obtained sequences matched the sequences in the targeted region in the *ITS* of *Cronobacter* spp. strains perfectly. Our results shown that the established LAMP method can detect *Cronobacter* spp. with high specificity using the primers with an appropriate label system.

### Sensitivity test in pure cultures

**Fig. 5:**
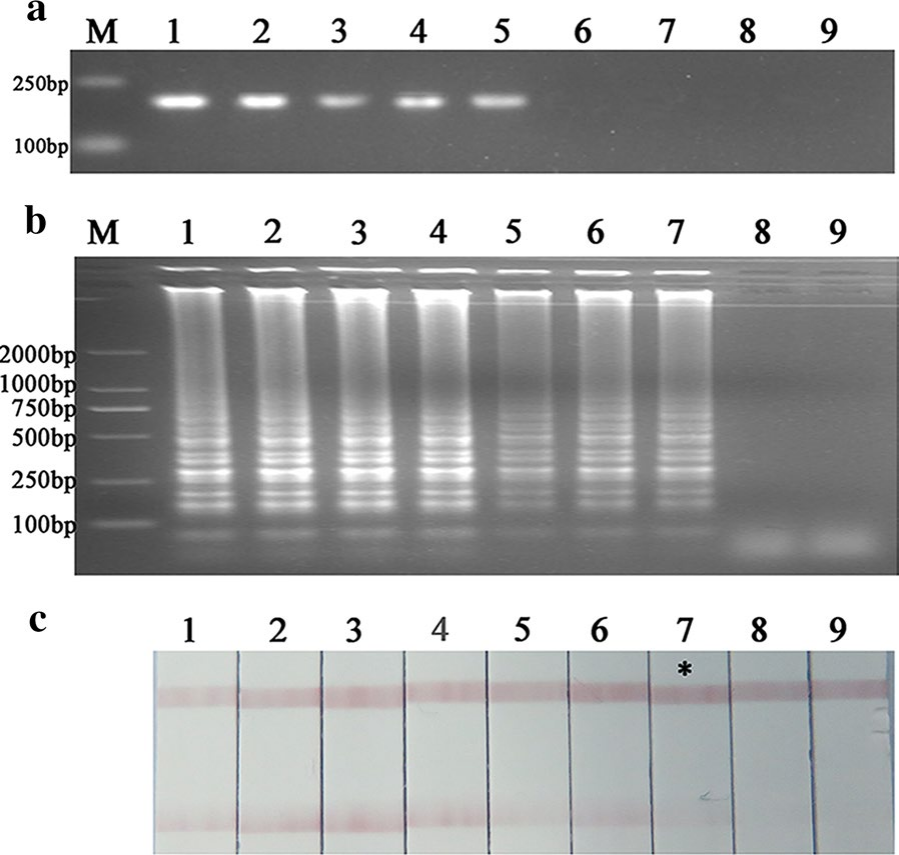
The sensitivity test of *Cronobacter* spp. in pure culture. **a** PCR; **b** loop-mediated isothermal amplification-agarose gel electrophoresis (LAMP-AGE); **c** loop-mediated isothermal amplification-lateral flow assay (LAMP-LFA). M: marker. Lanes 1 to 8: the concentrations of *Cronobacter sakazakii* ATCC 29544 ranging from 4.8 × 10^6^ to 4.8 × 10^−1^ cfu/mL; lane 9, negative control

4.8 × 10^6^ cfu/mL *C. sakazakii* through plate counts from ATCC 29544 was ten-fold diluted to a minimum concentration equivalent to 4.8 × 10^−1^ cfu/mL. DNA was efficiently extracted from each of *C. sakazakii* dilution. The detection limit of PCR assay was 4.8 × 10^2^ cfu/mL by AGE analysis, while that of the “Fast LAMP” was 4.8 × 10^0^ cfu/mL. Simultaneously, by the novel label LFA system analysis, the detectable red color in the test line was 4.8 × 10^0^ cfu/mL consistent with AGE analysis (Fig. 5). Neither of the results in the LAMP assay utilizing the nuclease-free water as negative control indicated a signal.

### Detection of *Cronobacter* spp. in PIF

**Fig. 6:**
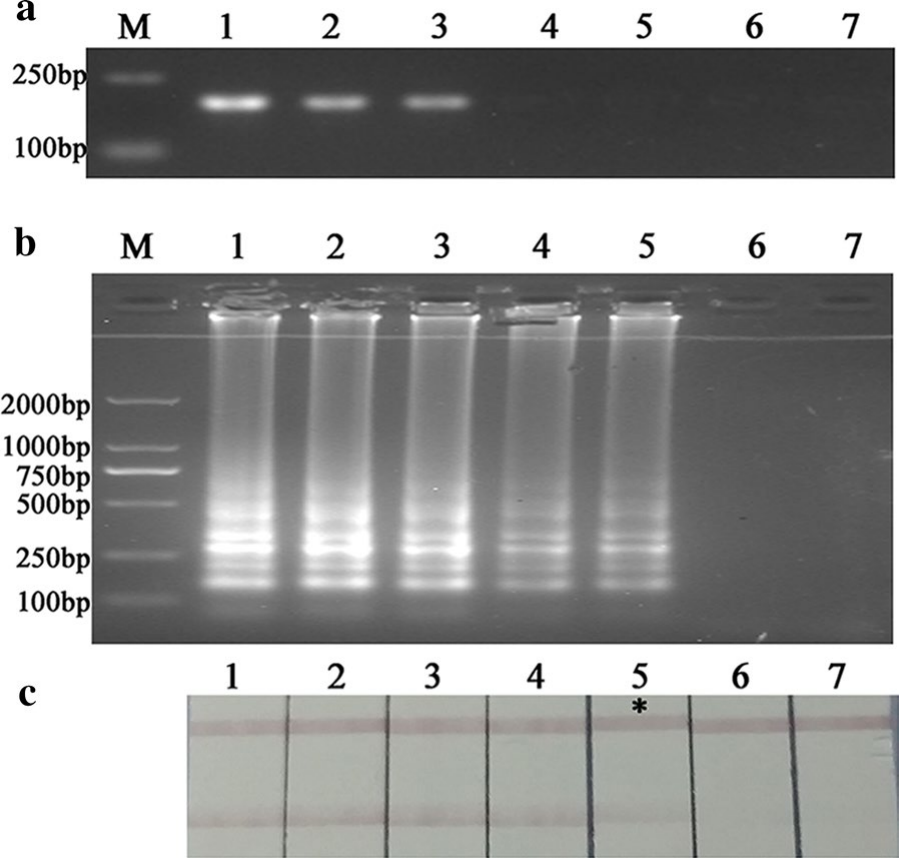
The sensitivity test of *Cronobacter* spp. in PIF. **a** PCR; **b** loop-mediated isothermal amplification-agarose gel electrophoresis (LAMP-AGE); **c** loop-mediated isothermal amplification-lateral flow assay (LAMP-LFA). M: marker. Lanes 1 to 6: the PIF contaminated with concentrations of the *Cronobacter sakazakii* ATCC 29544 ranging from 5.6 × 10^5^ to 5.6 × 10^0^ cfu/g. Lane 7, negative control

The sensitivity of detecting *C. sakazakii* ATCC 29544 in real PIF samples using the LAMP-LFA and LAMP-AGE was demonstrated at a limit of 5.6 × 10^1^ cfu/g (Fig. 6) without enrichment. However, in comparison the conventional PCR assay based on F3 and B3 primers only to a minimum concentration equivalent to that of 5.6 × 10^3^ cfu/g, indicating that the novel label-based LAMP-LFA system was 100 times more sensitive than PCR. Meanwhile, 6 *Cronobacter* species contaminated in PIF exhibited the same sensitivity in Table 5. No amplification observed in the negative control with each of the method.

**Table 5:** Detection limit of *Cronobacter* genus in PIF

Bacterial species	Detection limit (cfu/g)
*C. sakazakii*	5.6 × 10^1^
*C. malonaticus*	2.4 × 10^1^
*C. turicensis*	4.6 × 10^1^
*C. muytjensii*	7.9 × 10^1^
*C. universalis*	4.7 × 10^1^
*C. dublinensis*	1.9 × 10^1^
*C. condimenti*	9.2 × 10^1^

## Discussion

Rapid and accurate detection of *Cronobacter* spp. was essential to control the spread of *Cronobacter* spp. in PIF. This report which described the evaluation of the novel probe-free LAMP method combined with LFA was more specific than the conventional PCR to detect *Cronobacter* spp. in PIF. In our investigation, this novel and simple strategy for LAMP-LFA detection of *Cronobacter* spp. with the probe-free system was based on genetically variable and species-specific 16S–23S *ITS* rRNA. It was found that the primers designed based on the 16S–23S rRNA showed high specificity in this probe-free label system for the detection of *Cronobacter* spp. Liu et al. found similar results using LAMP with *ITS* sequence that all of the 15 strains of *Cronobacter* spp. showed positive results while all of the 61 strains of non-*Cronobacter* spp. were examined to be negative results (Liu et al. 2010). It illustrated that 16S–23S *ITS* rRNA to be a reliable sequence for the detection of *Cronobacter* species and can be utilized to discriminate *Cronobacter* spp. from all non-*Cronobacter* strains. Traditionally, the LAMP assay can be performed in 40–60 min to obtain favorable results based on *ompA* sequence, in which the detection limit in pure cultures was 10^2^ cfu/mL (Fan et al. 2012). Though this LAMP assay was free of betaine, the determined amplification time was reduced to 25 min compared to the previous amplification time. Our investigation will provide a new route to establish a LAMP assay for rapid detection.

LAMP amplicons were visualized in LFA based on the probe-label system. However, previous study demonstrated that sometimes the probe-dependent label system was time-consuming, complicated, and inaccurate (Najian et al. 2016). In addition, it may yield the false-positive results caused by the carry-over contamination when the probe labeled with FITC was added at the end of amplification. Removal of the step of probe hybridization can avoid the incubation step before detection analysis and minimize the detection time. Here the use of FITC to label the probe and the probe incubation step before the LFA analysis were eliminated. This novel label system avoided false positives induced by the carryover contamination, exhibiting high efficiency of our results. However, the non-specific amplification due to self-dimerization and hetero-dimer formation resulted in an error-prone data analysis, demonstrating that the negative results revealed false positive results. Briefly, the detection result could be positive for non-*Cronobacter* spp. sample without DNA template by using the LAMP-LFA method, this may be a key impediment in application.

Although the probe-free system was described and introduced into pathogenic detection in 2016 (Najian et al. 2016), the problem of primer dimer resulting in false positives was still ignored. There was no formation of primer dimer by analysis of the self-dimerization and hetero-dimer formation when 6 LAMP primers were employed to perform the LAMP assay. However, once the presence of primer dimer tagged with markers such as FITC and biotin in the LAMP–LFA assay, the false positive results would be observed. Essentially, the primer dimer was induced by the non-specific amplification, one of the particular problems known to cause inhibition of primer hybridization to the template and reduction of a number of primers available for annealing. Also, the probe-free label system was faced with the same challenge. It was demonstrated that the FITC based probe primer released the fluorescence signals not only in the specific isothermal amplification but also in the “primer dimer” caused by the non-specific amplification (Tian et al. 2014). Thus the reduction of primer dimer in the amplification assay is of great significance. Previously, Taq SSB protein was successfully used for enhancement of PCR amplification efficiency. It enables to combine proteins with ssDNA rather than dsDNA, thus preventing the production of the secondary-structure or dimer formation due to the primer mismatches. Here we revealed that the Taq SSB protein could be acted as a powerful reagent addictive to the LAMP amplification. Application of the Taq SSB protein to the LAMP assay can significantly improve the efficiency of this amplification assay. Simultaneously, the primer dimer can be reduced to rule out the false positive results in LFA. The detection sensitivity achieved by Taq SSB protein based fast-LAMP assay increased to 10^1^ cfu/g in the protein-rich PIF. Nevertheless, results also demonstrated that the excess Taq SSB protein in LAMP assay may occupy the site of the primers and prevent the primers binding with the dsDNA template for amplification. Therefore, the appropriate concentration of Taq SSB protein can be helpfully applied to the LAMP assay in the bio-diagnostics area. The novel label-based system took advantage of Taq SSB protein to bind with the large or small fragments to eliminate spurious results of LFA and consequently provided a new method in detection. Meanwhile, Taq SSB added to LAMP reaction replaced betaine and enhanced the efficiency of amplification.

Detection of *Cronobacter* spp. in the complex and protein-rich PIF is a painstaking problem in the detection area. Direct application of the LFA in detecting *Cronobacter* spp. will be innovative. However, a major challenge is the lack of highly specific antibodies, hindering the application in detecting *Cronobacter* spp. based on antibody strips. In this paper, the LFA combination with LAMP was found to be sensitive and specific in monitoring *Cronobacter* spp. in PIF, in which the components of the running buffer and blocking buffer were determined to stabilize the LFA and maintain the detection sensitivity consistent with the AGE analysis. Commonly, Tween-20 in LFA can promote the release of gold conjugates, avoid non-specific reaction on the pad and increase the intensity of the red line (Rivas et al. 2015). The addition of BSA and trehalose can enhance the specificity and efficiency of the LFA strips. Thus, the optimized LFA simplified the operation, avoided the use of carcinogenic substances (such as EB), and minimized the detection time. Our approach provided a practical method for the diagnosis of *Cronobacter* spp. in PIF. Finally, this investigation demonstrated that this fast LAMP assay combined LFA is the first report to demonstrate a probe-free label system for specific detection of *Cronobacter* spp.

The detection limit of LAMP-LFA based on *ITS* in real samples is 10^1^ cfu/g, ten times more sensitive than the past efforts (Hu et al. 2009; Fan et al. 2012). As suggested by the international microbiological standards that all species of *Cronobacter* must be absent in 10 g of PIF. Although in this study the minimum culture time for the detection of *Cronobacter* spp. with LAMP-LFA was not mentioned, the method of less than 6 h enrichment and overnight can be applied to achieve the desired sensitivity (Fan et al. 2012). Therefore, the novel probe-free LAMP-LFA has been demonstrated as a cost-effective, practical tool for detecting *Cronobacter* spp. in PIF.

In summary, our investigation revealed that the combination of the “Fast LAMP” method with LFA by probe-free and specific label system was proven to be a sensitive method to detect *Cronobacter* genus in PIF. Simultaneously, the LAMP amplicons directly double embedded with FITC and biotin can be specifically captured by LFA, in which the false positive results due to the primer dimers were efficiently eliminated by introducing the Taq SSB protein into the LAMP assay. And the total detection time of this system was within 40 min. We found that the sensitivity of this rapid LAMP-LFA system, which was consistent with LAMP-AGE, was 100 times higher than the traditional PCR method. It was 10^0^ cfu/mL in pure culture while that was 10^1^ cfu/g in PIF without enrichment, respectively. Therefore, this novel label-based LAMP-LFA with probe-free can be considered as a rapid, highly sensitive and specific tool for the detection of *Cronobacter* spp. in PIF, especially in limited resource settings. Simultaneously, this method could provide an advanced platform for multiplex detection in rapid detection field.

## Abbreviations

*Cronobacter* spp.: *Cronobacter* species
PIF: powdered infant formula
LAMP: loop-mediated isothermal amplification
AGE: agarose gel electrophoresis
LFA: lateral flow assay
EB: ethidium bromide
FITC: fluorescein isothiocyanate
SA: streptavidin
AuNPs: gold nanoparticles
SSB: single strand DNA binding
NC: nitrocellulose
